# Editorial: T cell exhaustion; mechanisms of induction, modulation, and recovery

**DOI:** 10.3389/fimmu.2022.1122530

**Published:** 2023-01-05

**Authors:** Mohamed S. Abdel-Hakeem

**Affiliations:** ^1^ Department of Pathology and Laboratory Medicine, and Emory Vaccine Center, Emory School of Medicine, Emory University, Atlanta, GA, United States; ^2^ Department of Microbiology and Immunology, Faculty of Pharmacy, Cairo University, Cairo, Egypt

**Keywords:** T cell exhaustion, Tex subsets, Tex reinvigoration, regulatory T cells (Tregs), exhaustion signature in CAD

## Introduction

The current definition of T cell exhaustion has been coined in the immunology literature since 2003 (1). Exhaustion describes the differentiation pathway of T cells subjected to persistent antigenic stimulation during chronic viral infection and cancer (2). Exhausted T cells (T_EX_) are characterized by sustained elevated expression of inhibitory receptors (IRs), associated with progressive hierarchical loss of effector functions (1). Thus, IR-blockade represented a promising strategy to reinvigorate T_EX_ (3). Indeed, checkpoint blockade revolutionized immunotherapy against cancer and cured patients resistant to classical therapeutic interventions (4).

Despite 20 years of profiling T_EX_ cellularly, functionally, and transcriptionally, it is only in recent years that studies characterized the epigenetic profile (5–7) and subset dynamics of T_EX_ (8–11), as well as the interplay of several transcription factors (TFs) in the exhaustion program (12–14). Deeper insights into T_EX_ biology would unravel novel immunotherapeutic strategies. Seven research manuscripts and reviews in this Research Topic shed light on several aspects of T cell exhaustion.

## Insights into T_EX_ subset dynamics from HCV

Hepatitis C virus (HCV) infection is unique as the only human chronic viral infection where ~20-25% of patients spontaneously resolve the infection during the acute phase (rHCV), whereas most patients develop chronic disease (cHCV) (15). Another unique aspect of HCV is that it is now curable using direct-acting antivirals with ~95% sustained virologic response (SVR) success rate (15). Wildner et al. show that the master TF regulating exhaustion, TOX, was expressed in CD8 T_EX_ across all disease stages and groups of patients, but at various levels. However, expression of another TF, eomesodermin (Eomes), was significantly higher in cHCV in T cells targeting unmutated epitopes compared to “off-target” T cells where the epitope underwent escape mutations inhibiting recognition by epitope-specific CD8 T cells. TOX-Eomes co-expression was associated with a more terminally differentiated phenotype of T_EX_ (PD1^hi^ CD39^hi^ CD73^lo^ CD127^–^) *versus* CD39^lo^ CD73^hi^ CD127^+^ phenotype characteristic of the progenitor T_EX_ subset. SVR patients showed a mixed phenotype, which retains the expression of Eomes and IRs, while expressing the memory associated markers CD127 and CD73 characteristic of rHCV. This opens a new venue for investigating the modulation of T_EX_ differentiation and subset dynamics by TOX-interactions with Eomes which is relatively understudied.

Immune pressure by T cells drives mutation of their cognate viral epitopes (15), however it is not known how this in return affects T_EX_ phenotype. Osuch et al. add another layer to factors shaping T_EX_ phenotype, as they show that epitope polymorphism within HCV impacts T_EX_ subset dynamics. Interestingly, for some epitopes (e.g. NS_1406_) variations from the genotype-specific consensus sequence were associated with higher frequency of more terminally differentiated PD1^+^Tim-3^+^ T_EX_, however for other epitopes (e.g. NS_1073_) it was the contrary. These findings highlight the importance of dissecting T_EX_ subset dynamics within the context of the autologous epitope sequences.

## T_EX_ reinvigoration; targeting PSGL-1, CD226, CD137, metabolism, and Tregs

As checkpoint blockade revolutionized immunotherapy, and the list of IRs keeps growing (16), more IRs are being targeted for T_EX_ reinvigoration. The discovery of the inhibitory effects of PSGL-1 on T_EX_ suggested immunotherapeutic potential (17). In this Research Topic, Viramontes et al. show that PSGL-1 modulates T_EX_ biology in multiple ways. Deleting PSGL-1-encoding gene (*Selplg* ^–/–^) confirmed its role in curtailing expansion of CD8 T_EX_, but at the same time showed it importance in maintaining virus/tumor-specific T cells, and the differentiation of the progenitor T_EX_ subset. This highlights the potential of targeting PSGL-1 as a promising immunotherapeutic strategy, which was demonstrated by the same group in combination with PD1-blockade in a mouse melanoma model (18).

Another important aspect of T_EX_ deviation from the acute differentiation path is adopting a different metabolic lifestyle (19), which was confirmed in consequent studies (20, 21). In the context of HIV, Alrubayyi et al. demonstrate that viremic individuals showed a more terminally differentiated phenotype of T_EX_ (PD1^hi^ Eomes^hi^ TIGIT^+^) compared to *Elite Controllers* who naturally controlled HIV viremia. Importantly, this was associated with higher expression of Glut-1 glucose transporter, impaired mitochondrial function, and mitochondrial biogenesis. Multipronged treatment with a scavenger of reactive oxygen species (ROS), pharmacological inhibitors of dysregulated mitochondrial respiratory capacity, and interleukin-15 (IL-15) that enhances mitochondrial biogenesis promoted partial functional restoration of HIV-specific T_EX_.

Two significant reviews address points that are not commonly tackled when discussing CD8 T cell exhaustion. Pichler et al. discussed targeting less-studied co-stimulatory molecules such as CD226 and CD137, and signaling pathways such as phosphoinositide-3 (PI3) kinase for modulating T_EX_. As well as novel methods to enhance the stem-like/progenitor TCF1^+^ T_EX_ subset which is the main subset responding to immunotherapy (22). McRitchie and Akkaya focus on one of the extrinsic factors affecting CD8 T cell exhaustion, which is CD4 regulatory T cells (Tregs). They specifically elaborate on the implication of Tregs within the tumor microenvironment (TME) on T_EX_, and the factors that promote the migration and immunosuppressive functions of Tregs.

## T_EX_ beyond chronic infection and cancer

An important implication of T_EX_ is their role in the pathology of pharmacological diseases other than cancer or chronic viral infection. One example is atherosclerosis, the main underlying cause of coronary artery disease (CAD) (23, 24). Zhao et al. use computational biology tools, such as gene set variation analysis (GSVA), to identify hub genes associated with CD8 T cells in the context of CAD, that are shared with T_EX_ signatures from multiple cancers. This identified biomarkers for CD8 T cells associated with CAD, and highlights the importance of monitoring T_EX_ features for CAD.

## Conclusion

Overall, the articles in this Research Topic highlight the pivotal role of T_EX_ in a wide range of diseases and underscore the importance of deciphering more aspects of T_EX_ biology and the factors modulating the exhaustion program. This is pivotal to inform the design of next generation immunotherapies. The majority of articles highlighted the importance of dissecting the underlying T_EX_ subset dynamics when studying these aspects and factors.

## Author contributions

The author confirms being the sole contributor of this work and has approved it for publication.
